# Wnt4 and LAP2alpha as Pacemakers of Thymic Epithelial Senescence

**DOI:** 10.1371/journal.pone.0010701

**Published:** 2010-05-18

**Authors:** Krisztian Kvell, Zoltan Varecza, Domokos Bartis, Sebastian Hesse, Sonia Parnell, Graham Anderson, Eric J. Jenkinson, Judit E. Pongracz

**Affiliations:** 1 Department of Medical Biotechnology, Institute for Immunology and Biotechnology, University of Pecs, Pecs, Hungary; 2 Division of Immunity and Infection, Department of Anatomy, Institute for Biomedical Research, University of Birmingham, Birmingham, United Kingdom; New Mexico State University, United States of America

## Abstract

Age-associated thymic involution has considerable physiological impact by inhibiting *de novo* T-cell selection. This impaired T-cell production leads to weakened immune responses. Yet the molecular mechanisms of thymic stromal adipose involution are not clear. Age-related alterations also occur in the murine thymus providing an excellent model system. In the present work structural and molecular changes of the murine thymic stroma were investigated during aging. We show that thymic epithelial senescence correlates with significant destruction of epithelial network followed by adipose involution. We also show in purified thymic epithelial cells the age-related down-regulation of Wnt4 (and subsequently FoxN1), and the prominent increase in LAP2α expression. These senescence-related changes of gene expression are strikingly similar to those observed during mesenchymal to pre-adipocyte differentiation of fibroblast cells suggesting similar molecular background in epithelial cells. For molecular level proof-of-principle stable LAP2α and Wnt4-over-expressing thymic epithelial cell lines were established. LAP2α over-expression provoked a surge of PPARγ expression, a transcription factor expressed in pre-adipocytes. In contrast, additional Wnt4 decreased the mRNA level of ADRP, a target gene of PPARγ. Murine embryonic thymic lobes have also been transfected with LAP2α- or Wnt4-encoding lentiviral vectors. As expected LAP2α over-expression increased, while additional Wnt4 secretion suppressed PPARγ expression. Based on these pioneer experiments we propose that decreased Wnt activity and increased LAP2α expression provide the molecular basis during thymic senescence. We suggest that these molecular changes trigger thymic epithelial senescence accompanied by adipose involution. This process may either occur directly where epithelium can trans-differentiate into pre-adipocytes; or indirectly where first epithelial to mesenchymal transition (EMT) occurs followed by subsequent pre-adipocyte differentiation. The latter version fits better with literature data and is supported by the observed histological and molecular level changes.

## Introduction

### Thymic senescence

Thymic senescence begins early, around late puberty. This process is called adipose involution, as the thymus is invaded by adipose tissue [1]. Due to decrease in thymic epithelial tissue mass, the thymus can no longer support the same output of T-cell production [2]. Therefore peripheral blood T lymphocyte composition exhibits the dominance of memory T lymphocytes resulting in impaired responses towards novel, particularly viral infections [3], [4], [5]. Since the thymic epithelium has a key role in deleting auto-reactive T-cell clones, functional impairment increases the chances of developing auto-immune disease [6]. If we were able to slow down or even stop the loss of thymic epithelium the elderly would have a better chance to address late-onset autoimmune diseases and viral infections. However, despite studies of thymic senescence, the molecular mechanism of thymic aging remains elusive.

### Signaling pathways of thymic epithelial cell development and maintenance

Understanding signaling mechanisms that regulate tissue development and maintenance of thymic epithelial cells might reveal the process of adipose involution. Certainly, maintenance and functional integrity of the thymic stroma requires stimuli through Notch, BMP, and Wnt signaling pathways [7], [8], [9], [10], [11]. Undoubtedly, the Wnt family of secreted glycoproteins is one of the best analyzed among the required ligands [12]. Most members of the nineteen known Wnt glycoproteins have been implicated in both the development of embryonic thymus and the maintenance of adult thymic epithelium [13]. In the thymus, Wnt ligands originate primarily from thymic epithelial cells and activate a highly complex signaling network via ten G-protein dependent receptors called Frizzleds (Fz), and their co-receptors of low-density lipoprotein receptor-related proteins 5/6 called LRP5/6 [14], [15]. The actual constellation of ligands, receptors, co-receptors and further regulatory molecules define Wnt-mediated effects. Recent studies have highlighted Wnt4 as responsible for the direct up-regulation of FoxN1, a key transcription factor responsible for the differentiation of thymic epithelial cells and the subsequent maintenance of thymic epithelial identity [13]. Interestingly, the Wnt/β-catenin pathway is known to efficiently block the adipocyte differentiation program in mesenchymal elements like fibroblasts [16], [17], [18], [19].

### Trans-differentiation of fibroblasts into adipocytes

Studies with fibroblast cells have also revealed that fibroblast to pre-adipocyte transformation is strongly connected to LAP2α, the member of the LAP2 protein family [17]. To date there are 7 classified intranuclear LAP2 polypeptides marked by the Greek alphabet. They are all splice variants of the same LAP2 gene previously called thymopoietin. While most splice variants associate with the nuclear envelope, LAP2α is involved in several nucleoplasmic activities including cell-cycle control and differentiation [20], [21]. LAP2α is synthesized in the cytoplasm and is then transported into the nucleus by a PKC-dependent mechanism [22]. The mere over-expression of LAP2α in fibroblasts is known to directly up-regulate PPARγ expression, an acknowledged marker and key transcription factor of pre-adipocyte differentiation [17]. In pre-adipocytes PPARγ expression is followed by an increase of ADRP expression (adipose differentiation-related protein) a known direct target gene of PPARγ. Although LAP2α over-expression alone initiates pre-adipocyte differentiation in fibroblasts, it is not sufficient to complete the adipocyte differentiation program in the absence of additional stimuli [17].

## Results and Discussion

### Disintegration of epithelial network

**Figure 1:**
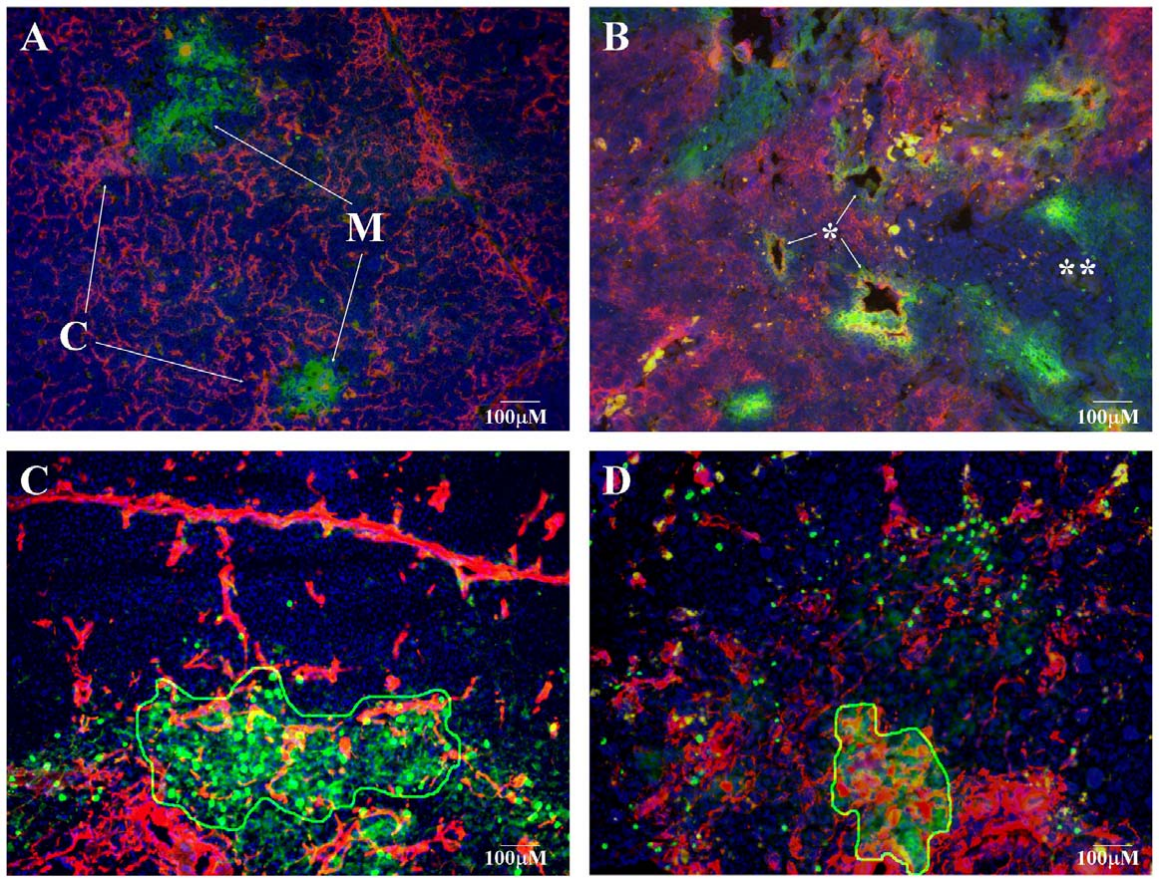
Disintegration of epithelial network. Figure 1A demonstrates cryostat section of 1 month, whereas figure 1B presents cryostat section of 1 year old BALB/c mouse thymus. Staining pattern: anti-EpCAM1-FITC (green), anti-Ly51-PE (red), DAPI (blue). ‘M’ marks medullary (EpCAM1^++^, Ly51^−^), while ‘C’ marks cortical (EpCAM1^+^, Ly51^++^) epithelial compartments on Figure 1A. Single asterisk (*) marks degenerative vacuoles, while double asterisk (**) mark the loss of epithelial staining on Figure 1B. Figure 1C (lower left) shows cryostate section of 2 month, whereas figure 1D (lower right) demonstrates cryostate section of 9 month old BALB/c mouse thymus. Staining pattern: anti-EpCAM1-FITC, ER-TR7-PE, DAPI (blue). The EpCAM1^++^ thymic medulla is outlined by continuous line on Figures 1C and 1D for easier visualization.

Senescence exhibits characteristic histological changes in both the human and mouse thymus [1], [23]. In order to demonstrate this process the thymic lobes of 1 month and 1 year old BALB/c mice were analyzed (see Figures 1A and 1B). In young adult mice, histology revealed strict segregation of epithelial cell compartments by staining for medullary (EpCAM1^++^, Ly51^−^) and cortical (EpCAM1^+^, Ly51^++^) epithelial cellular subsets (Figure 1A). This shows high level of morphological integrity just preceding puberty/early adulthood. However, the highly organized structure disintegrates and becomes chaotic by the age of 1 year (Figure 1B). By this age the previously shown strict cortico-medullary delineation becomes disintegrated, degenerative vacuoles appear surrounded by areas showing strong co-staining with both epithelial markers. There are also other large cellular areas that lack staining with either epithelial marker, a pattern completely absent at the young adult age.

Staining of extracellular matrix components of fibroblast origin (ER-TR7^++^) was also performed on cryostate thymic sections of 2 month and 9 month old BALB/c mice to identify epithelial and mesenchymal elements in young adult and aging thymic lobes. The above ages were selected to check additional time points and more precisely map the timeframe of thymic physiological senescence (see Figures 1C and 1D). The staining patterns are strikingly different at the two ages examined. In the 2 month old thymic tissue section a-EpCAM1 and ER-TR7-staining show little tendency for co-localization. In stark contrast, by the age of 9 months a-EpCAM1 and ER-TR7-staining show significant overlap within the thymic medulla, a phenomenon completely absent at earlier ages.

### Adipose involution

**Figure 2:**
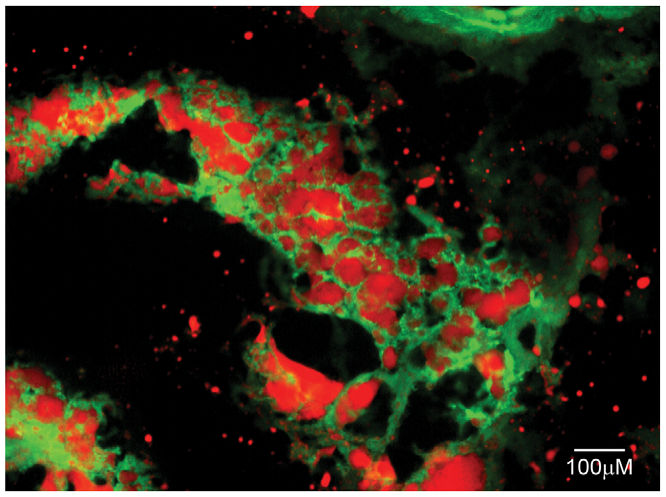
Adipose involution. Figure 2 shows adipose involution over cryostat section of 1.5 year old GFP-transgenic BALB/c mouse thymus. Staining pattern: GFP (green), LipidTox Red (red).

To demonstrate how the disorganization of thymic epithelial network is followed by the emergence of adipocytes, thymic sections of 1.5 year old GFP-transgenic BALB/c mice were analyzed. This mouse strain develops and reproduces exactly like control BALB/c mice, and the thymic epithelial function and thymocyte maturation is indistinguishable from wild type controls [24]. However, due to the ubiquitous and strong EF1 promoter-driven transgene transcription, bright GFP expression offers a native, green-colored, cytoplasmic staining for all the cells in these mice. Thymic sections of senescent GFP-transgenic mice were co-stained with LipidTox Red to identify adipocytes. Histology shows the presence of relatively large, inflated cells in which the green-colored (GFP-containing) cytoplasm is pushed to the periphery by red-staining neutral lipid deposits, a pattern characteristic of adipose cells (see Figure 2).

### Molecular changes of thymic epithelium

**Figure 3:**
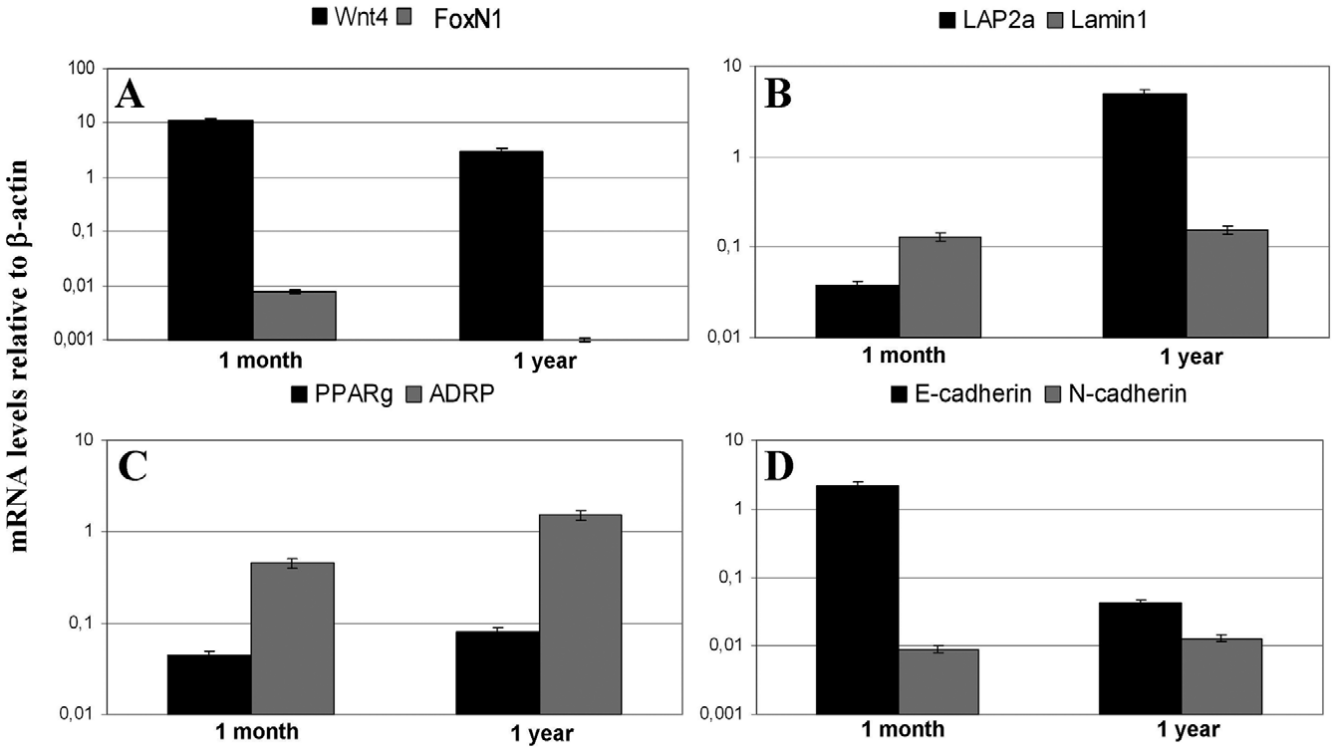
Molecular changes in thymic epithelium. Figures 3A–D demonstrate gene expression changes of MACS purified thymic epithelial cells measured by Q-PCR. Please note that the Y-axis scale is logarithmic. Error bars show ±1 SD.

Having presented structural changes of thymic epithelial senescence, we set out to investigate the underlying molecular events. In order to detect gene expression changes, thymic epithelial cells were purified from 1 month and 1 year old BALB/c mice based on EpCAM1 expression (MACS separation). Following cDNA synthesis, quantitative RT-PCR analysis was performed. Several genes including Wnt4, FoxN1, PPARγ, ADRP, lamin1 and LAP2α were tested (Table 1 lists primer sequences and characteristics, see Figures 3A–D for changes in gene expression). Figure 3A shows that the expression of both Wnt4 and FoxN1 decreases in thymic epithelial cells. Highly decreased level (or total absence in some cases) of FoxN1 could be the consequence of strong Wnt4 down-regulation by the age of 1 year, indicating that thymic epithelial cells can down-regulate FoxN1 expression while maintaining that of epithelial cell surface markers like EpCAM1 [13]. At the same time, mRNA levels of pre-adipocyte differentiation markers PPARγ and ADRP rise with age in the same, EpCAM1-positive cell population (Figure 3C). This finding is in harmony with histological data demonstrating the emergence of adipocytes in the thymic lobes of senescent mice (Figure 2). The expression of lamin1, a key component of the nuclear lamina remains unaffected during senescence in thymic epithelial cells; whereas, the expression of LAP2α increases significantly (see Figure 3B). This degree of dissociation between lamin1 and LAP2α expression is of note and suggests functional differences despite conventionally anticipated association of lamin1 and LAP2 molecular family members. The measured LAP2α up-regulation associated with age-related adipose involution is, however, in perfect agreement with other literature data suggesting the pre-adipocyte differentiation-promoting effect of LAP2α in fibroblasts [17]. This is the first report to show that such, normally fibroblast associated molecular changes occur in purified thymic epithelial cells. In the literature, epithelial-mesenchymal transition is associated with differential expression of E- and N-cadherin [25]. While E-cadherin decreases, N-cadherin normally compensates for the loss of E-cadherin expression. To investigate whether the first step towards pre-adipocyte differentiation is the epithelial-mesenchymal transition of epithelial cells, gene expression changes of E-cadherin and N-cadherin were measured (Figure 3D). While E-cadherin mRNA levels significantly decreased, N-cadherin gene expression showed a slight increase, indicating that EMT might be the initial step in epithelial cell transition to become pre-adipocytes.

**Table 1:** List of gene specific PCR primers.

Gene	Forward primer	Reverse primer
**β-actin**	5′-TGG CGC TTT TGA CTC AGG A -3′	5′-GGG AGG GTG AGG GAC TTC C - 3′
**Wnt4**	5′-CTC AAA GGC CTG ATC CAG AG - 3′	5′-TCA CAG CCA CAC TTC TCC AG - 3′
**LAP2α**	5′-TGA ACT GCA GGC AGC TAA GA-3′	5′-TCA TAG CTA GAC TCT GAG G-3′
**Lamin1**	5′ - TGA GTA CAA CCT GCG CTC AC -3′	5′ - TGA CTA GGT TGT CCC CGA AG -3′
**PPARγ**	5′ - CCC AAT GGT TGC TGA TTA CAA A -3′	5′ - AAT AAT AAG GTG GAG ATG CAG GTT CT -3′
**ADRP**	5′ - CGC CAT CGG ACA CTT CCT TA -3′	5′ - GTG ATG GCA GGC GAC ATC T -3′
**E-cadherin**	5′- AAG TGA CCG ATG ATG ATG CC -3′	5′- CTT CAT TCA CGT CTA CCA CGT -3′
**N-cadherin**	5′ - GTG GAG GCT TCT GGT GAA AT - 3′	5′ - CTG CTG GCT CGC TGC TT - 3′
**FoxN1**	Applied Biosystems TaqMan probe PN4351272 (Mm00477457_m1)	

### Transgenic cell lines

**Figure 4:**
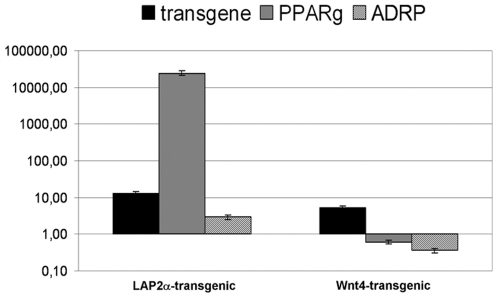
Confirmation in transgenic thymic cell lines. Figure 4 shows gene expression changes of LAP2α and Wnt4 over-expressing transgenic TEP1 cells measured by Q-PCR. Please note that Y-axis scale is logarithmic. Error bars show ±1 SD.

Stable LAP2α over-expressing or Wnt4-secreting transgenic TEP1 cell lines were established using lentiviral transgenesis. The use of a primary-derived model cell line provides the advantage of absolute purity, the complete lack of other cell types that could potentially affect the gene expression profile of epithelial cells [26]. The established transgenic cell lines proliferated normally and did not show obvious signs of phenotypic changes (data not shown). In contrast to morphology, quantitative RT-PCR analysis revealed that LAP2α over-expression triggers an immense surge of PPARγ expression (Figure 4). Such an increase in mRNA level suggests that this is not a plain quantitative, but rather a qualitative change. ADRP a direct target gene of PPARγ was also up-regulated albeit to a lesser extent (Figure 4). On the other hand in Wnt4-secreting TEP1 cells the mRNA level of both PPARγ and ADRP was decreased (Figure 4). In the TEP1 cell line the expression of FoxN1 could not be addressed as it is very low/undetectable and remains as such with all the tested treatments (data not shown).

### Transfected embryonic thymic organ cultures

**Figure 5:**
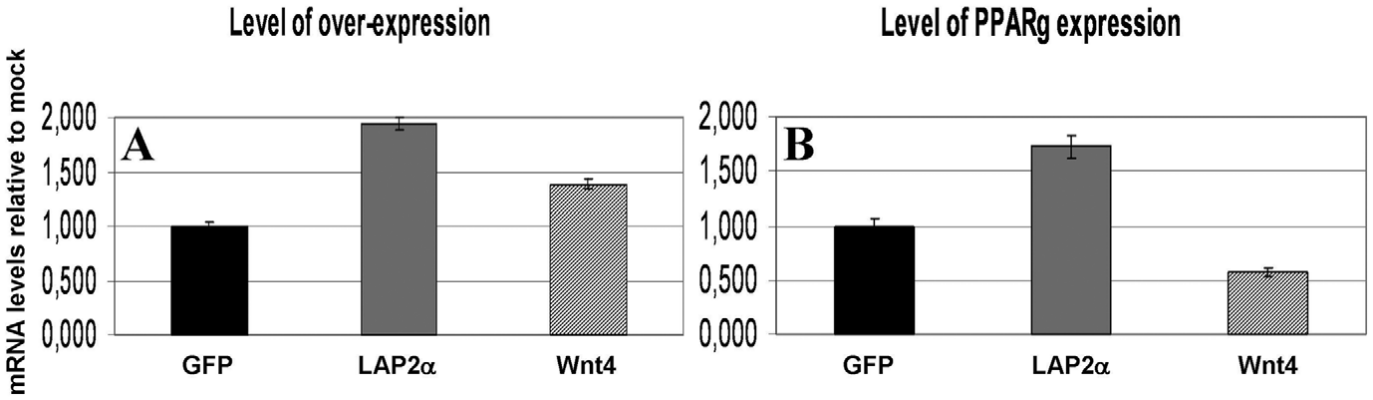
Confirmation in transfected thymic lobes. Figures 5A–B present gene expression changes measured by Q-PCR from cDNA of murine thymic lobes transfected at E12 and cultured for 4 days *in vitro*. Please note that Y-axis scale is linear. Error bars show ±1 SD.

To confirm the involvement of LAP2α and Wnt4 during adipogenesis through their direct effect on PPARγ expression in primary cells, murine thymic lobes were isolated from timed pregnancies at E12. Thymic lobes at the age of E12 provide an excellent experimental setting where the thymus has just been formed and there is no sign of aging. Furthermore, thymic lobes at this stage are also small enough to be both cultured and transfected as a whole, nutrients and virions have free access to most of the cells in the lobe without the need of disrupting any intercellular connection or tissue matrix [8], [27]. The isolated lobes were therefore transfected with lentiviral vectors encoding GFP (mock), Wnt4 or LAP2α and were cultured for 4 days *in vitro*. Q-PCR was performed to confirm over-expression of LAP2α and Wnt4 in the embryonic thymic lobes as a result of lentiviral transgenesis (Figure 5A) and their effect on PPARγ expression was also analyzed (Figure 5B). The level of over-expression was confirmed following transfection with both LAP2α- and Wnt4-encoding viral vectors. Q-PCR analysis revealed that LAP2α over-expression triggers an increase of PPARγ expression, whereas additional Wnt4 secretion suppresses PPARγ level (Figure 5B). The latter Wnt4-mediated suppression of PPARγ expression in cultures of E12 thymic embryonic lobes was also confirmed by treatment with Wnt4-containing supernatants of Wnt4 over-expressing TEP1 cell line (data not shown). Interestingly, the expression of FoxN1 did not decrease in LAP2α over-expressing thymic lobes (data not shown), possibly due to high levels of Wnt4 in the embryonic thymic tissue preserving FoxN1 status. Our molecular studies using E12 thymic lobes confirmed our data obtained with the TEP1 cell lines, that even in embryonic thymic tissue pre-adipocyte differentiation markers can be up-regulated in the presence of LAP2α, indicating that the process can be dissected and controlled at a molecular level.

### Conclusion

Here we show that with senescence, thymic epithelial Wnt4 secretion decreases, possibly below a threshold level that is required to maintain the identity of established thymic epithelial cells. This is measured by the loss of FoxN1 expression, a key transcription factor defining thymic epithelial cell identity. However, these epithelial cells still express cell surface markers characteristic for thymic epithelial cells – i.e. EpCAM1. Wnt4 deprivation opens up an opportunity for trans-differentiation into pre-adipocytes. The simultaneous increase in LAP2α expression provides the necessary signal that pushes de-differentiated thymic epithelial cells to differentiate into pre-adipocytes, as detected by increased mRNA levels of PPARγ and ADRP.

**Figure 6:**
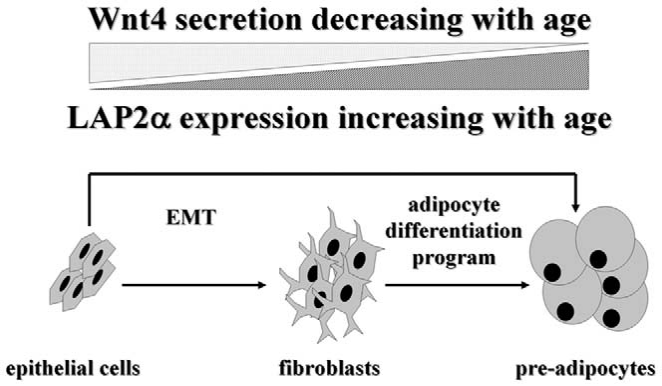
Model for thymic epithelial senescence. Figure 6 demonstrates our molecular level model of thymic adipose involution. Decreasing Wnt4 and increasing LAP2α levels promote epithelial cells to differentiate into pre-adipocytes either directly or indirectly via EMT.

We propose two different mechanisms for the process of adipose involution (see Figure 6). The first allows for the direct initiation of pre-adipocyte differentiation from de-differentiated thymic epithelial cells due to the down-regulation of Wnt4 and up-regulation of LAP2α. Although we cannot rule out this first model, we favor the second model where the process occurs indirectly: de-differentiation of thymic epithelial cells triggers EMT first, and then the resulting fibroblasts undergo the conventional route of differentiation program towards adipocyte-lineage commitment. The latter model certainly fits better with current literature of EMT [28] and is also supported by our histological and molecular results. Co-localization of a-EpCAM1 and ER-TR7-staining in the aging thymic medulla (Figure 1D) confirms that in the 9 month old thymus there are cells expressing the EpCAM1 marker as a legacy of their primary origin, and also secreting ER-TR7-positive extracellular matrix components, a function conventionally attributed to fibroblast cells. Moreover, Q-PCR data obtained with cDNA samples of MACS-purified thymic epithelial cells also demonstrate an age-related shift in cadherin expression levels characteristic for EMT (Figure 3D) providing additional evidence for the active process of EMT during thymic epithelial senescence.

Our model of thymic epithelial senescence is based on data obtained with mice undergoing physiological senescence. This is the first model for the molecular basis of the thymic epithelium to undergo adipose involution. This model withstands molecular level proof-of-principle using both a model cell line and primary embryonic thymic organ cultures rendered transgenic by lentiviral transgenesis.

### Perspectives

Further experiments, however, are required. We plan using inducible, LAP2α-transgenic mice to allow us precise temporal-spatial over-expression of LAP2α in adult thymic epithelium to model and decisively verify the role of LAP2α in pre-adipocyte trans-differentiation *in vivo* exploiting our experience in establishing transgenic animals [24], [29]. If LAP2α proves to be a master regulator of thymic adipose involution *in vivo* too, this knowledge appoints LAP2α as target molecule for directed rejuvenation of the thymic epithelial structure and function. This rejuvenation process could theoretically reinforce naïve T-cell output to reach young adult levels that could ameliorate senescence-related immunological disorders like impaired antiviral defense and late-onset auto-immune diseases.

## Methods

### Cell lines and mice

The 293T (ATCC: CRL-11268) and TEP1 [26] cell lines were cultured in DMEM supplemented with 10% FCS, penicillin, streptomycin and β-mercapto-ethanol (Lonza Walkersville). For the experiments we used thymic lobes from timed pregnancies at E12, and also from adult BALB/c mice at 4 week and 1 year of age, and from 1.5 year old GFP-transgenic BALB/c-mice. Mice were bred in our animal facility; all animal work has been conducted according to relevant national and international guidelines following approval of ethics committee of the University of Pecs. Senescent animals developed and aged normally, without any treatment.

### Transgenic cell, organ and animal models

The GFP-transgenic BALB/c model was created using lentiviral transgenesis as published by our group [24]. The Wnt4 sequence was purchased and subcloned from an Origene (Origene) vector containing human full-length Wnt4 cDNA. The full-length murine LAP2α cDNA containing plasmid was a kind gift of Dr. Simon Amos. The GFP (mock), LAP2α or Wnt4 over-expressing TEP1 cell lines or E12 thymic lobes were generated using lentiviral vectors that were prepared as described previously [30]. Following overnight lentiviral transfection the thymic lobes were transferred over Nucleopore Track-Etch Membranes (Whatman) and were cultured in DMEM supplemented with 20% FCS, penicillin, streptomycin, ciprofloxacin, amphotericin-B and β-mercapto-ethanol (Lonza Walkersville).

### Histology using fluorescent antibodies, proteins and dyes

Sections (9 µm) of frozen thymic lobes of BALB/c mice were fixed in cold acetone, then dried and blocked using 5% BSA in PBS for 20 min before staining with a-Ly51-PE (clone 6C3), a-EpCAM-FITC (clone G8.8), ER-TR7-PE antibodies and DAPI. Thymic sections of GFP-transgenic mice were fixed in 4% paraformaldehyde before staining with LipidTOX Red following the manufacturer's instructions (Invitrogen). The sections were analyzed using an Olympus BX61 microscope equipped with a CCD camera and AnalySIS software.

### Separation and enrichment of thymic epithelial cells

Thymic lobes were digested with type F collagenase from *C. hystolyticum* (Sigma) for 30 min, then washed with DMEM 10% FCS. Cell suspensions were then labeled with anti-EpCAM1-FITC (clone G8.8) and washed with MACS-buffer followed by incubation with anti-FITC micro-beads (Miltenyi Biotec), the EpCAM^+^-cells were used for total RNA isolation and subsequent quantitative PCR analysis. The cells were purified using MACS LS separation columns (Miltenyi Biotec).

### RNA isolation, preparation of cDNA, Q-PCR analysis

Total RNA was isolated the RNAII kit (Macherey-Nagel), including an on column DNA digestion step. cDNA was constructed using the high capacity RNA to cDNA kit (Applied Biosystems). For Q- PCR analysis, we used an AB7500 platform and either SYBR green or TaqMan PCR master mix (Applied Biosystems). Gene expression was normalized to β-actin. The sequences and data of primers are listed in Table 1.

### Statistical analysis

All experiments were performed on three occasions, representative experiments are shown. Measures were obtained in triplicates; data are presented as mean ±1 SD by error bars.
